# To Evaluate the Relationship between Mandibular Canine Calcification Stages and Skeletal Age

**DOI:** 10.5005/jp-journals-10005-1127

**Published:** 2012-02-24

**Authors:** Pooja Malik, Vivek Rana, Usha Rehani

**Affiliations:** Senior Lecturer, Department of Pedodontics, Kalka Dental College Meerut, Uttar Pradesh, India, e-mail: dr_pooja_malik@hotmail.com; Professor, Department of Pedodontics, Subharti Dental College Meerut, Uttar Pradesh, India; Professor, Department of Pedodontics, ITS Dental College, Greater Noida, Uttar Pradesh, India

**Keywords:** Demirjian’s method, Greulich-Pyle method, Skeletal age, Canine calcification stages

## Abstract

**Aim:** The purpose of this study was to assess skeletal age and establish relationship between mandibular canine calcification and skeletal age.

**Materials and methods:** The study included 147 females aged 10 to 13 years. The subjects were divided into three groups: Group I—comprising of 10 to 11 years old female; Group II—comprising of 11 to 12 years old female; Group III—12 to 13 years female. OPG and hand and wrist radiographs of left side for each subject were taken with prior consent of their parents. The calcification status of canine was evaluated from orthopantomograms according to scores given in Demirjian’s method. The stages of ossification of various carpal bones were evaluated using radiographic atlas of Greulich-Pyle and skeletal age was calculated.

**Results:** Data collected was statistically analyzed.

**Conclusion:** The results drawn from this study showed that a strong correlation was observed for canine calcification stage F for 10 to 11 years and for stage G in 11 to 12 years and 12 to 13 years respectively.

**How to cite this article:** Malik P, Rana V, Rehani U. To Evaluate the Relationship between Mandibular Canine Calcification Stages and Skeletal Age. Int J Clin Pediatr Dent 2012;5(1): 14-19.

## INTRODUCTION

Growth and development are two important parameters of human life. Growth is the base from which development emerges. It is a dynamic process with a stable pattern of changes resulting in the increase in physical size and mass during its course of development.

Dental age, chronological age, skeletal age are all essential parameters that help to assess the growth and development in children. In somatic growth and development, it is already an accepted fact that girls are more advanced than boys, up to the preadolescent years. During the growing years, it is observed that girls are usually 1 to 6 months ahead of boys. As far as the dentition is concerned, the difference in the eruption of the canines can be as great as 11 months in some populations. For this reason dental development has been accepted as a better maturity indicator than emergence.^1^

Assessment of skeletal maturity is an important method in the evaluation, follow-up and timing of therapy in children with growth disorders, such as constitutional growth retardation and growth hormone deficiency, as well as endocrinological diseases, such as hypothyroidism, congenital adrenal hyperplasia and precocious puberty.

Dental maturity can be determined by the stage of tooth eruption or by the stage of tooth formation.^2,3^ Tooth formation is proposed as a more reliable criterion for determining dental maturation. The ease of recognition of dental development stages, together with the availability of periapical or panoramic radiographs in most orthodontic or pediatric dental practices are practical reasons for attempting to assess the physiologic maturity without resorting to hand- wrist radiographs.

If a strong association exists between skeletal maturity and dental calcification stages, the stages of dental calcification might be used as a first-level diagnostic tool to estimate the timing of the pubertal growth spurt. In the literature, interrelationship between skeletal, somatic and sexual maturity have been shown to be consistently strong.

The technique for assessing skeletal maturity consists of visual inspection of the developing bones, their initial appearance and their subsequent ossification-related changes in shape and size. Various areas of the skeleton have been used: The foot, ankle, hip, elbow, hand-wrist and cervical vertebrae.^4^ The hand-wrist radiograph is commonly used for skeletal developmental assessment. One of the most frequently applied methods to estimate skeletal age is the atlas of Greulich and Pyle.^5^

Interestingly, the correlation between calcification stages of individual teeth and skeletal maturity has also been reported previously. Garn et al showed only weak correlations between third molar and skeletal development, whereas Engström et al reported stronger relationships. Racial variations in the relationship have also been suggested.^6^Mappes et al indicated that the predominant ethnic origin of the population, climate, nutrition, socioeconomic levels, and urbanization are causative factors of these racial variations.

Relationships between the stages of tooth mineralization of the mandibular canine appear to correlate better with ossification stages than do the other teeth.^2,5,6^

Growth assessment parameters as well as several other anthropometric measurements are useful in the inter- disciplinary team evaluation of patient with various types of short stature, endocrine and/or metabolic disease, syndrome identification and forensics.^7^

Therefore, this study is being conducted for independent verification, and to establish canine development as a valid clinical tool for growth estimation, in the benefit of patients.

## MATERIALS AND METHODS

The study was conducted at the Department of Pedodontics and Preventive Dentistry, Subharti Dental College, Meerut. The sample consisted of 147 orthopantomograms of the teeth and hand and wrist radiographs from girls randomly selected from various areas of Meerut from the age group of 10 to 13 years known chronologic age.

The criteria for selection of cases for the present study were as follows:

1. The subject should be clinically free from any developmental endocrine or nutritional disorder as this may affect development of a subject.
2. Subject should be clinically free from any past prolonged illness.

**Fig. 1:**
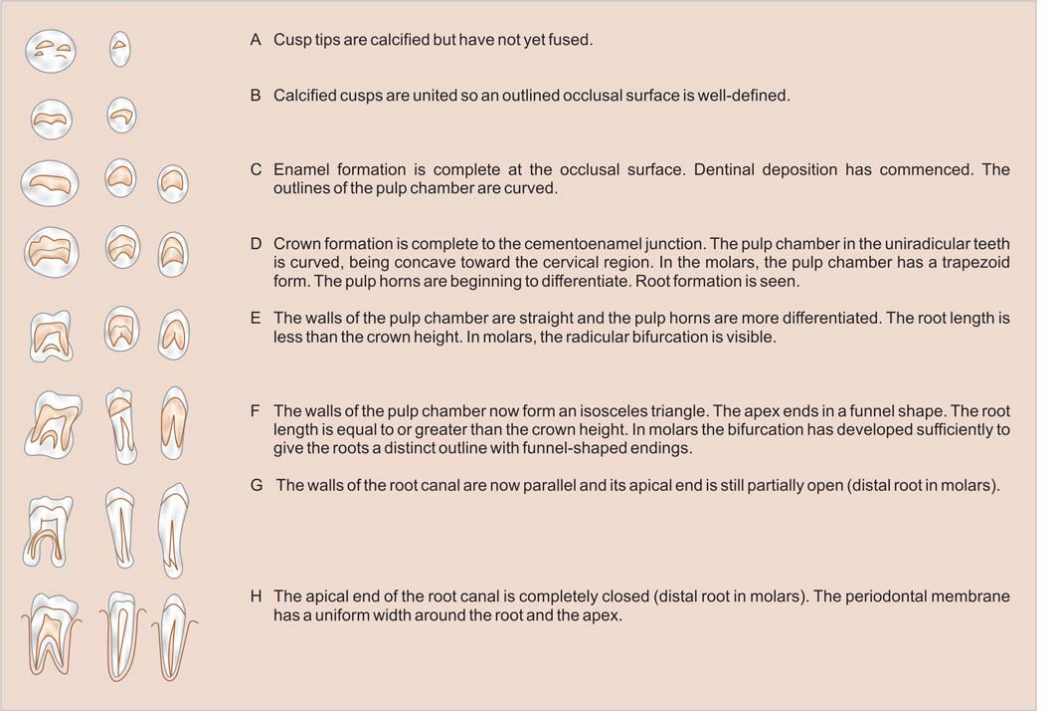
Tooth calcification stages from A to H

**Fig. 2:**
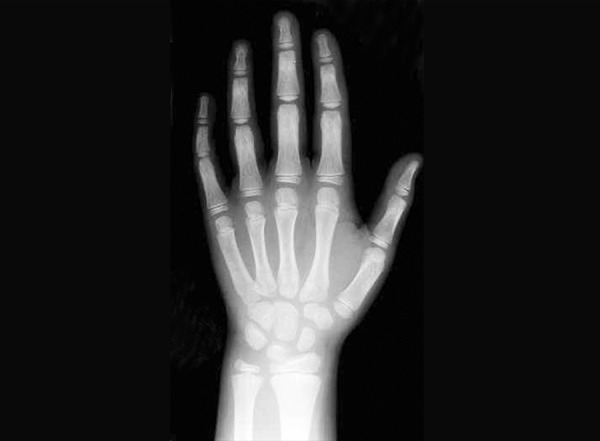
Hand and wrist radiograph showing carpal bones

The study was carried out in following steps:

- Brief history of every child was taken (according to proforma) who fulfills the selection criteria, which includes child’s name, age and sex, date of birth, father’s name, address and school. Date of birth of each child was checked from school records.
- Dental examination was done with a probe, mouth mirror, and tweezer under good illumination and the state of eruption of teeth was seen.
- Informed consent was taken.
- The orthopantomograms were viewed on X-ray viewer. The stage of calcification of left permanent mandibular canine teeth was seen according to Demirjian’s method (Fig. 1) and recorded on the proforma.
- Radiograph of hand and wrist of left side was taken on 8 × 10" X-rays films (Kodak) by seating the individual on an adjustable stool in front of the table, so that the child places the forearm along a line parallel to the shoulders. The cassette was placed with its long axis parallel to the long axis of the hand and exposed for 0.04 seconds to X-radiation at 46 KVP and 100 mA.
- Hand and wrist radiograph was viewed on a view box and the state of ossification of various carpal bones were seen (Fig. 2) and recorded on the proforma.

**Figure:**
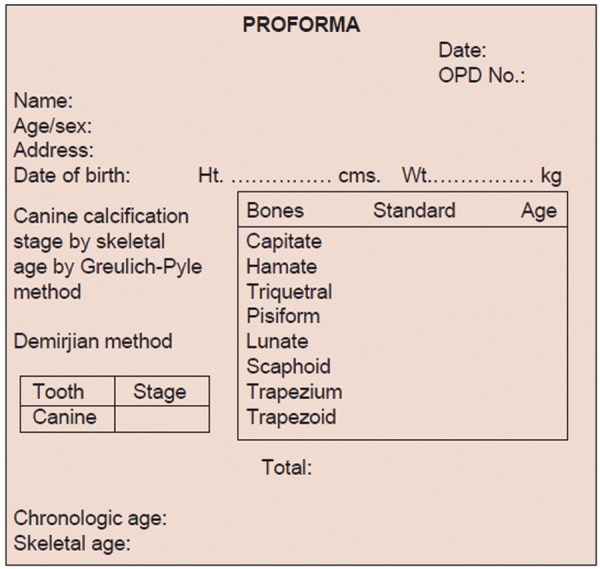


Radiographs for each subject were taken in the department of oral medicine and radiology. All the exposed films were developed, fixed and dried.

The stages of mandibular canine calcification and skeletal age were calculated as follows:

### Assessment of Stage of Calcification of Mandibular Permanent Canine—Demirjian’s Method

Method

According to method given by Demirjian, stage of calcification of mandibular canine is assessed by the radiological appearances of the tooth. Each tooth has been rated according to developmental criteria (amount of dentinal deposit, shape change of pulp chamber, etc.) rather than changes in size. Eight stages, A to H, have been defined from the first appearance of calcified points to the closure of the apex (Fig. 1).

### Skeletal Age Assessment—Greulich and Pyle Method

Method

The skeletal age by hand-wrist radiograph was assessed by evaluating the stages of ossification of various carpal bones. Bones were then analyzed following a standardized sequence in which they usually appear: Capitate, hamate, triquetral, lunate, scaphoid, trapezium, trapezoid, pisiform (Fig. 2). In our study, skeletal age was calculated from standards given to each bone according to the criteria given in ‘radiographic atlas’ of Greulich and Pyle.

Finally, data collected was statistically analyzed.

## STATISTICAL ANALYSIS

Data was collected and divided into three groups for statistical analysis.

Group I: 10 to 11 years

Group II: 11 to 12 years

Group III: 12 to 13 years

Statistical analysis was carried out to evaluate the relationship between mandibular canine calcification stages and skeletal age. The descriptive data included mean and standard deviation for each group. Karl-Pearson’s correlation coefficient was used to correlate the values. Mandibular canine calcification stage was compared with skeletal age.

The statistical analysis was done by using SPSS version 16.5 software.

## RESULTS

**Graph 1:**
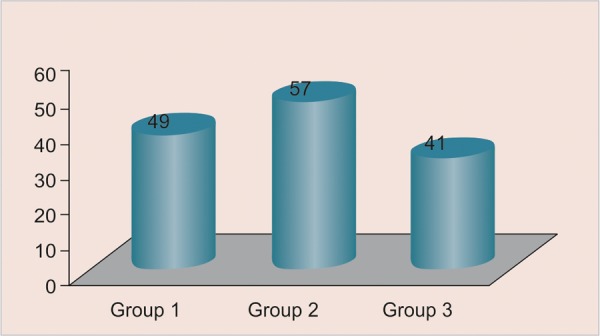
Distribution of subjects

Our study comprised a total of 147 females in the age group of 10 to 13 years. Distribution of subjects according to age is shown in Graph 1.

**Graph 2:**
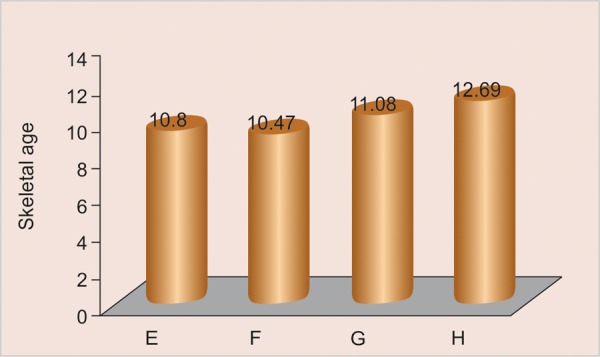
Distribution of skeletal age according to canine calcification stages in 10 to 11 years

**Graph 3:**
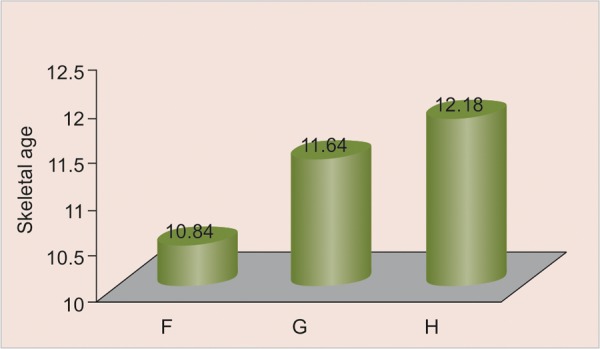
Distribution of skeletal age according to canine calcification stages in 11 to 12 years

**Graph 4:**
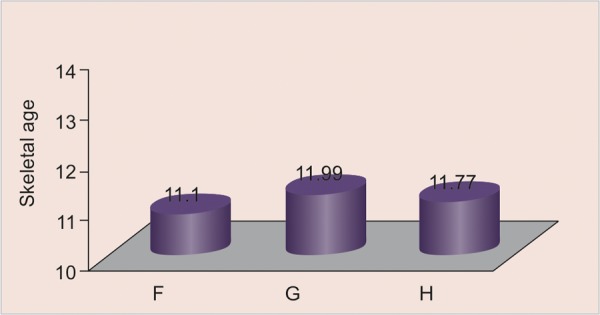
Distribution of skeletal age according to canine calcification stages in 12 to 13 years

All the values of the skeletal age are distributed according to canine calcification stages. Distribution of sample according to age groups 10 to 11, 11 to 12, 12 to 13 has been done as shown in Graphs 2 to 4 respectively.

All the values of the canine calcification stages according to the skeletal age are expressed in the form of mean ± SD (Graphs 2 to 4) for the age group 10 to 11 years, 11 to 12 years and 12 to 13 years respectively.

By applying Z-test to test the significant difference between the canine calcification stages for different age group, a significant difference was observed between canine calcification stages F-G, G-H, H-F in 10 to 11 years (Table 1) and for G-H, F-H in 11 to 12 years (Table 2) only at 5% level of significance.

In 12 to 13 years age group, there is no significant difference between canine calcification stages (Table 3).

**Table 1:** **Table 1: **Significant values between canine calcification stages in 10 to 11 years

*Group*		*Z_cal_*		*Z_tab_ (at 5%)*		*p-value*	
FG		2.220*		1.96		<0.05	
GH		21.19*		1.96		<0.05	
FH		19.91*		1.96		<0.05	

*Significant difference

**Table 2:** **Table 2: **Significant values between canine calcification stages in 11 to 12 years

*Group*		*Zcal*		*Z_tab_ (at 5%)*		*p-value*	
FG		1.870		1.96		>0.05	
GH		2.060*		1.96		<0.05	
FH		3.150*		1.96		<0.05	

*Significant difference

**Table 3:** **Table 3: **Significant values between canine calcification stages in 12 to 13 years

*Group*		*Z_cal_*		*Z_tab_ (at 5%)*		*p-value*	
FG		1.1055		1.96		>0.05	
GH		0.3920		1.96		>0.05	
FH		1.360		1.96		>0.05	

No significant difference

**Table 4:** **Table 4: **Correlation observed between skeletal age and canine calcification stages in different age groups

*Age group* *Skeletal age and canine stage*		*10-11*		*11-12*		*12-13*
r_Fs_		0.4927		0.3718		0.5921
r_Gs_		0.1562		0.4102		0.6798
r_Hs_		0.2870		0.2912		0.5882

**Graph 5:**
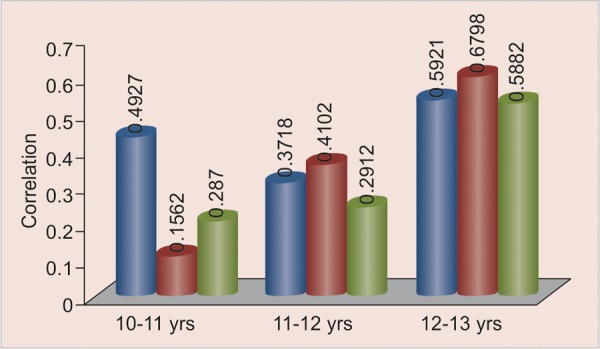
Correlation between canine calcification stages and skeletal age in 10 to 11, 11 to 12, 12 to 13 years

## DISCUSSION

Knowledge of maturation status of a child plays an important role in the diagnosis, treatment planning and eventual outcome of the treatment.^8^

Many researchers have attempted to determine whether there is a relationship between the level of skeletal maturity (SA) and the maturation of the permanent dentition. Nolla in 1960, reported dental eruption to be more variable than the calcification sequence in the dentition.^5^

Skeletal age can be assessed by several methods, but simplicity, convenience, and speed have made the Greulich and Pyle method the most commonly used standard for assessing skeletal maturation.^9^

The Greulich-Pyle method of skeletal age assessment is used widely; even in our study this method was used. Due to orderly sequence of formation, the hand-wrist is taken as one of the most reliable method of skeletal age assessment. To overcome the disadvantages of hand-wrist radiographs, mainly the additional exposure to the patient, researchers diverted their mind to determine skeletal maturation from those clinical records that are routinely used for orthodontic diagnosis and treatment planning. The most commonly used diagnostic record is the orthopantomogram.

Hassel and Farman (1995)^6^ developed cervical vertebrae as maturity indicators by assessing the lateral profile changes of second, third and fourth vertebrae. The cervical vertebrae maturity indicators were evaluated against skeletal maturation index established from hand-wrist radiograph by Fishman.

Several methods for the determination of dental development from radiograph have been described. The method basically observes the stage of mineralization of teeth observed in radiographs and code them accordingly to previously determined scores.

Evaluation of tooth maturation using the method of Demirjian and Goldstein is thought to be of great value.

Hagg and Matsson (1985)^10^ compared the reliability of three different methods for the assessment of dental maturity and concluded that the method described by Demirjian and Goldstein offered a high degree of reliability and precision.

The present study was undertaken to determine skeletal age and evaluate the relationship between skeletal age and mandibular canine calcification stages. Mandibular canine calcification stages are determined by Demirjian’s method. Female children of age 11 to 13 years who had no growth disorders were taken to avoid any irregularity in the results, as abnormal or delayed growth can have a significant effect on the dental as well as the skeletal age as stated by Gulati et al.^11^

Sandra Coutinho et al (1993)^7^ in their study assessed the relationships between mandibular canine calcification stages and skeletal maturity. Their results show close association between mandibular canine calcification stages and skeletal maturity indicators. Canine stage F indicates the initiation of puberty. The timing of stage G coincides with the capping of the third middle and the fifth proximal phalanges and the presence of the adductor sesamoid. The intermediate stage between stages F and G should be used to identify the early stages of the pubertal growth spurt. Dental calcification stages of the mandibular canine provide readily available and easily recognized indications of the maturity status of a person; they are simple first-level, diagnostic tests to determine whether additional, more sensitive, measures of maturity are warranted.

Sahar taher (2001)^12^ conducted a study to assess cervical vertebrae and mandibular canine calcification as skeletal maturation indicator. Highly significant relationship was found between all the various cervical maturation and mandibular canine calcification stages.

The methods of bone age determination are to some extent subjective, and may therefore be subjected to intraobserver and interobserver variability. This confirms the findings of King et al that bone age estimation improves with clinical experience.^13^

Results of our study also show strong correlation between canine calcification stages and skeletal age in 11 to 13 years females. Presence of canine calcification stage F shows proximity of skeletal age between 10 to 11 years, and stage G shows skeletal age proximity of 11 to 13 years.

The purpose of our investigation is to provide the pedodontist with an additional tool to help determine growth potential in the adolescent patient. By using a routinely taken diagnostic radiograph, i.e. orthopantomograms, we the pedodontist would have a reliable diagnostic tool to aid in formulating treatment options and to reduce radiation exposure to the patient simultaneously.^4^

An additional explanation may be that the present sample can be increased in the reference study. No two individuals grow and develop at the same rate. An understanding of growth events is of primary importance of clinical pediatric dentistry. Growth is a dynamic process involving change yet commonly the growth of a child is judged using reference data for attained status at an age.^14^

Further studies are needed with extensive and large number of samples in order to minimize the error. It might be better and would permit a more objective diagnostic evaluation if skeletal age is considered as a basis to help formulate a treatment plan.

## CONCLUSION

A total of 147 female subjects ranging from 10 to 13 years of age were included in the study. Brief history was taken.

Canine calcification stage was calculated by Demirjian’s method and skeletal age was recorded by Greulich and Pyle atlas.

Following conclusions were drawn from this study:

1. A strong correlation was observed for canine calcification stage F for 10 to 11 years and for stage G in 11 to 12 years and 12 to 13 years respectively.
2. Significant association was observed between the canine calcification stages and skeletal age for different age groups.
3. Significant difference was observed between canine calcification stages F-G, G-H, H-F in 10 to 11 years and for G-H, F-H in 11 to 12 years.
4. In 12 to 13 years age group, there is no significant diffe- rence between canine calcification stages.

Further studies are needed with extensive and large number of samples in order to minimize the error.

It might be better and would permit a more objective diagnostic evaluation, if skeletal age is considered as a basis to help formulate a treatment plan.
